# 555 The Art of Caring

**DOI:** 10.1093/jbcr/irac012.183

**Published:** 2022-03-23

**Authors:** Kristen C Quinn, Nan McCoy, Mary A Strawn, Callie M Thompson, Giavonni M Lewis

**Affiliations:** University of Utah Health, SLC, Utah; The Walter Hive, Scottsdale, Arizona; The Walter Hive, Phoenix, Arizona; University of Utah Health, Salt Lake City, Utah; University of Utah Burn Center, Salt Lake City, Utah

## Abstract

**Introduction:**

Providing Burn Care is a physically and emotionally demanding job. AND, it is a rewarding and inspiring profession. 2020 and 2021 saw unprecedented rates of burn out and turn over in staff. Our concern for the well being of staff as well as our need to increase retention motivated our desire to provide staff with tools to build resiliency and coping strategies. Traditionally, this education comes in the form of teaching mindfulness, breathing exercises or self care outside of the burn center. We recognized that while these approaches are important and effective, they are not the right fit for everyone. The current times require us to use a multipronged approach to the tools we offer our burn teams.

Our regional burn center partnered with a community based arts education nonprofit to re-think how we provide staff with tools for adaptability. A curriculum was formulated that provided therapeutic art to nursing staff via four weekly zoom meetings. This resulted in a novel approach to resiliency building and self care.

**Methods:**

The program was opened to all burn center staff members. Staff registered in advance for the four week program; the arts organization mailed each participant a box containing all the supplies they would for need. Each week, a different art modality/artist was paired with therapeutic discussions grounded in a trauma informed approach. Discussions focused on emotional and physical safety, building a community of support, finding balance between work and home and identifying coping skills to use to help ground ourselves during times of stress.

**Results:**

Seven burn center staff members signed up for this program. Weekly attendance averaged 6-7. A program evaluation was completed by participants at the end of the final session. Questions were divided into three sections: program logistics, community building and the artistic process. Results indicated that this was an extremely valuable experience for participants. In addition, staff made comments such as "allowing myself to be vulnerable in the process has built trust and connection between myself and the other participants", "I don't remember the last time I did something like this just for myself", "I feel like I can add art to my list of coping strategies, despite never thinking of myself as an artist".

**Conclusions:**

Offering burn center staff with an alternative program to help build resiliency resulted in an increased sense of trust between staff members, provided one more outlet for self care and allowed participants to build resiliency in a new way.

